# A case of primary COVID-19 pneumonia: plausible airborne transmission of SARS-CoV-2

**DOI:** 10.1186/s40001-022-00668-1

**Published:** 2022-04-04

**Authors:** Nathan Dumont-Leblond, Caroline Duchaine, Marc Veillette, Visal Pen, Marco Bergevin

**Affiliations:** 1 Centre de Recherche de l’Institut Universitaire de Cardiologie et de Pneumologie de Québec, Quebec, QC Canada; 2 Département de Biochimie, Université Laval, de microbiologie et de bio-informatique, Faculté des sciences et de génie, Quebec, QC Canada; 3 Canada Research Chair On Bioaerosols, Quebec, QC Canada; 4 Department of Medical Imaging, Cité-de-La-Santé Hospital, Laval, QC Canada; 5 Department of Microbiology, Cité-de-La-Santé Hospital, QC Laval, Canada

**Keywords:** SARS-CoV-2, COVID-19, Airborne transmission, Test, Diagnosis, Aerosols, Bioaerosols

## Abstract

**Background:**

The different clinical manifestations, from none to severe, and the variability in efficacy of SARS-CoV-2 diagnosis by upper respiratory tract testing, make diagnosis of COVID-19 and prevention of transmission especially challenging. In addition, the ways by which the virus can most efficiently transmit still remain unclear.

**Case Presentation:**

We report the case a 48-year-old man who presents primary COVID-19 pneumonia. He was initially admitted for cholecystitis but, upon review of his abdominal CT scan, a segmental zone of ground glass opacity was identified in the right lower lobe. A bronchoalveolar lavage proved positive to SARS-CoV-2 by RT-qPCR, even if he tested negative by oro-nasopharyngeal swab at admission and the day after he underwent bronchoscopy. The near absence of the virus in his saliva 2 days after, combined with a very sharp increase in salivary viral load on the third day, also rule out the possibility of prior viral replication in the upper airway and clearance. In addition, rapidly increasing bilateral alveolar lung infiltrates appeared as the upper respiratory tests begin to detect the virus.

**Conclusions:**

For this patient to have developed primary COVID-19 pneumonia, a contagious aerosol must have traveled to the lower respiratory system. This case gives indirect but compelling evidence that aerosol may spread the virus. It also highlights the limitations of oral and nasal testing methods and the importance of anatomical considerations when studying infections by SARS-CoV-2.

**Supplementary Information:**

The online version contains supplementary material available at 10.1186/s40001-022-00668-1.

## Background

Patient screening, testing, and backward contact tracing, remain important tools to limit the transmission of SARS-CoV-2 and to control the COVID-19 pandemic. However, the efficacy of these strategies relies on the use of appropriate technology and on proper understanding of the infection mechanisms at play. The standard nasopharyngeal and oral SARS-CoV-2 tests do not always allow diagnosis in infected patients. Early reports from China and Italy had warned of false negative nasopharyngeal and oropharyngeal swabs and the added value of the chest computed tomography scan or bronchoalveolar lavage [1–4].

The most widely adopted diagnostic test has nonetheless been nasopharyngeal or oro-nasopharyngeal swab. However, saliva sampling [5–8] and oropharyngeal gargling [9, 10] have been explored. These studies have highlighted that a minority of patients may test positive using one method but not the other. It has become clear that viral reproduction kinetics can vary by anatomic sites and throughout the course of infection. A predominant hypothesis explaining negative upper respiratory tract (URT) RT-qPCR with confirmed lower respiratory tract (LRT) infection has been the previous migration of the virus from the upper respiratory tract to the lower respiratory tract followed by clearing of the upper respiratory virus by the time of swabbing [2, 11].

On the other hand, the relative importance of the different routes of transmission of the illness is still under debate and so is the influence of symptoms and upper airway test positivity on an individual’s potential to transmit the virus. Yet, it is now acknowledged that SARS-CoV-2 can be transmitted by air from particles of a variety of sizes, encompassing what is traditionally defined as droplets and aerosols [12]. The respiratory tract depth at which airborne particles are produced influences their size distribution [13–15]. Different behaviors such as coughing, speech, and breathing also shape this distribution. Since the size of virus-bearing particles affects their potential to reach certain parts of the airways [16], it seems reasonable to think that it could influence the localization of the primary site of impaction and viral replication. Therefore, multiple scenarios of transmission could occur, including primary URT infections and primary LRT infections.

Here we report a case that highlights that discordant results from URT and LRT testing can occur in the context of primary COVID-19 pneumonia, implying respirable aerosol acquisition of the virus in the community.

## Case description

A 48-year-old male with untreated mild diabetes presented to the emergency room of Cité de la Santé hospital (Laval, Canada) on the 30th of November 2020, complaining of one week of ongoing diffuse abdominal pain and constipation. He did not complain of nausea, vomiting or diarrhea nor did he report any respiratory symptoms. He denied any contact with known COVID-19 cases either in his household or at the sushi restaurant where he worked. Due to lockdown measures, the restaurant was only open for takeout and the patient never served customers directly. It is also worth noting that no SARS-CoV-2 variants of concern were circulating in Quebec at that time.

**Fig. 1:**
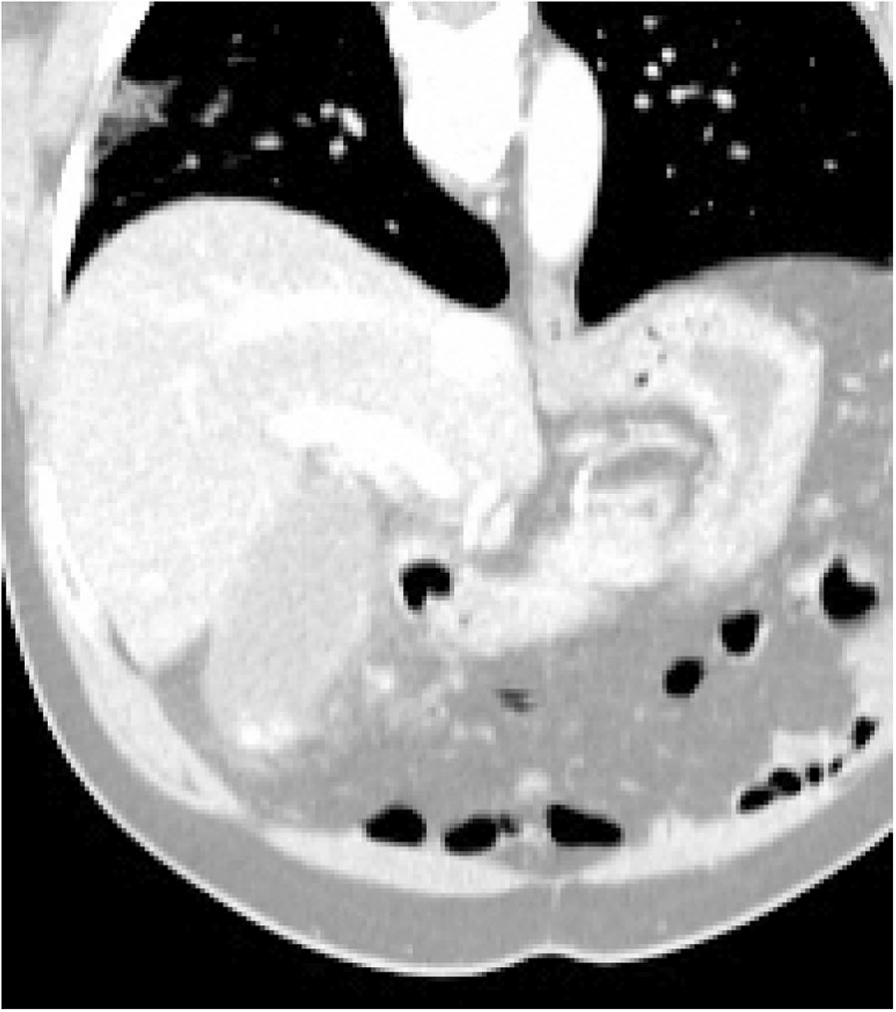
Abdominal CT scan. The image shows fat infiltration around a distended gallbladder that has wall thickening and stones, as well as a subsegmental ground glass opacity in the right lower lobe

**Fig. 2:**
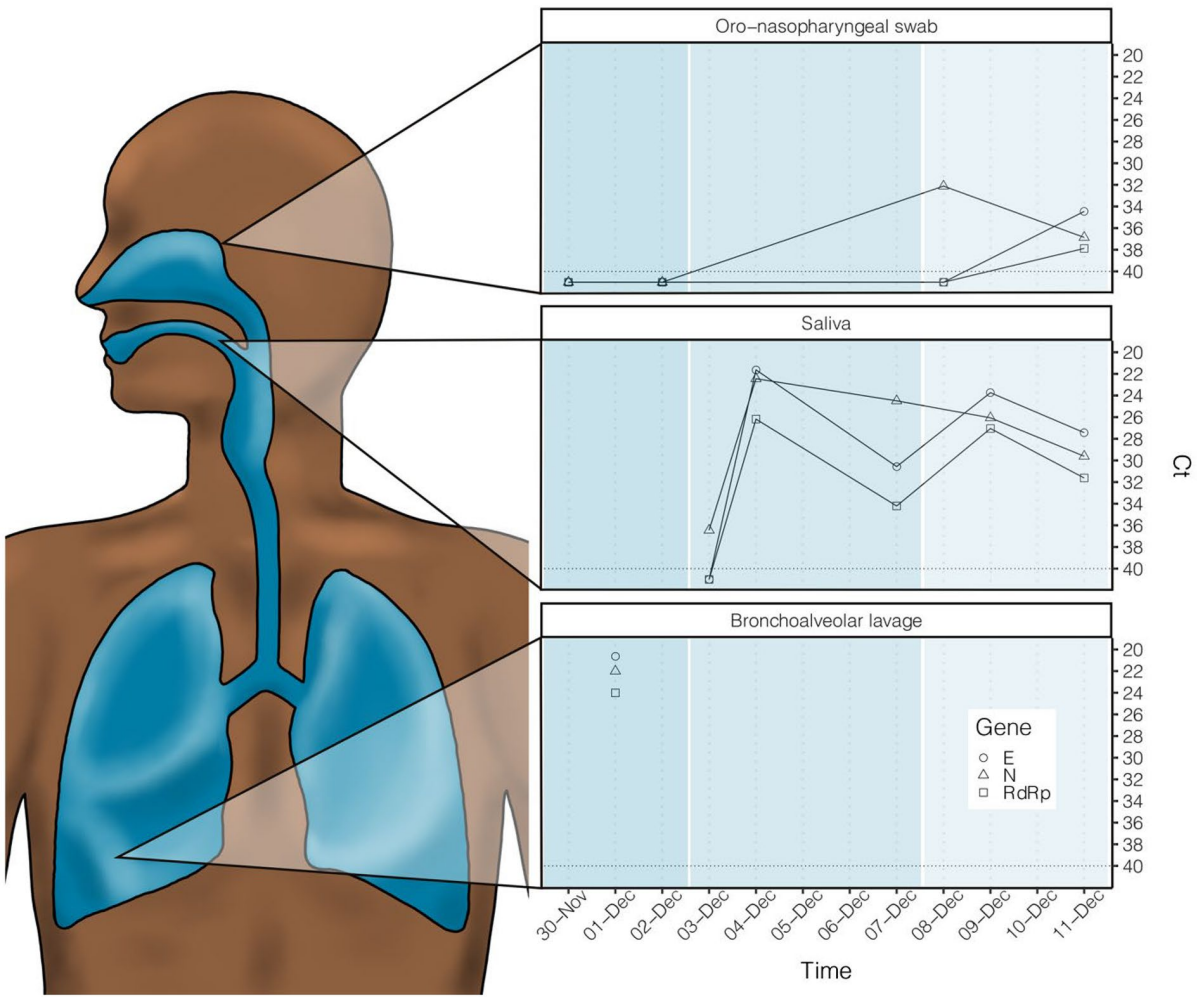
Quantification values in time from the three different sites sampled. Viral loads are expressed in Ct values. The shapes represent the different SARS-CoV-2 genes targeted

**Fig. 3:**
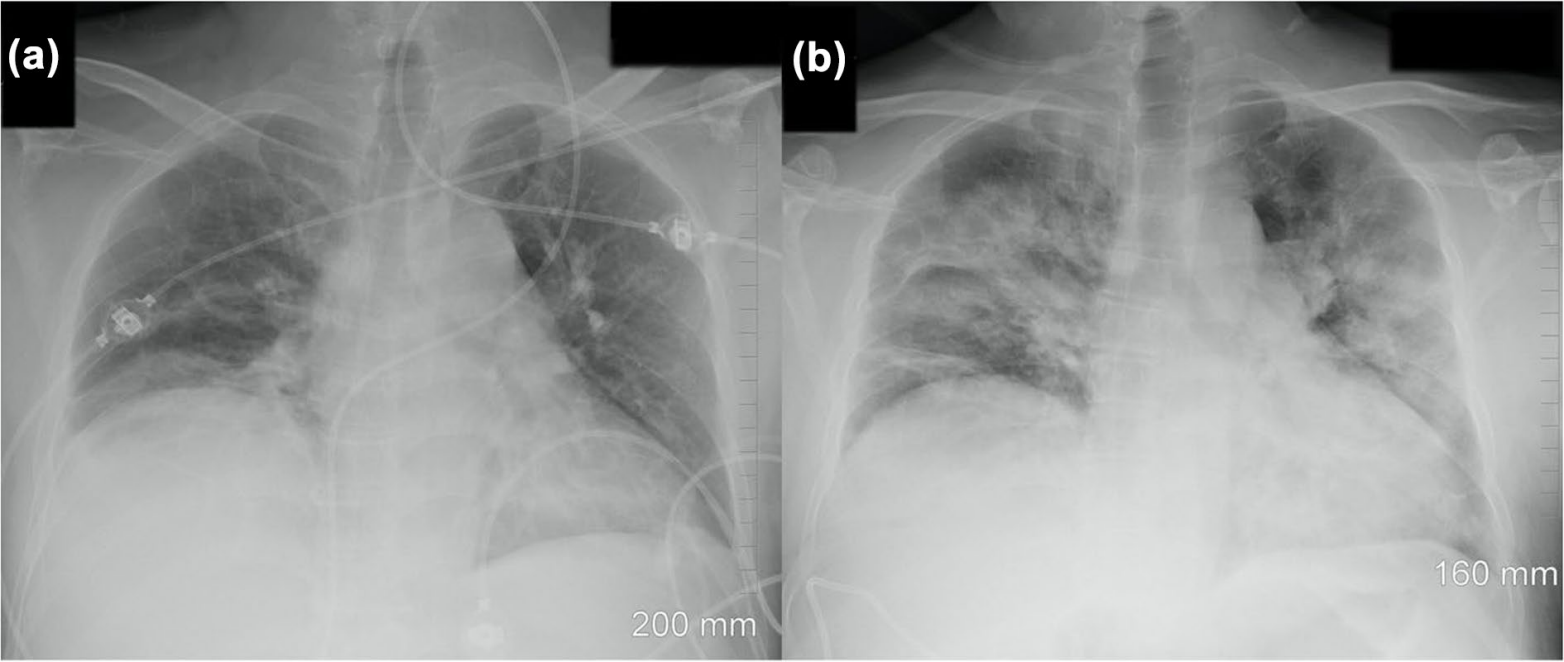
Chest radiographs demonstrate rapidly increasing alveolar lung infiltrates bilaterally. On December 2nd, the chest X-ray **a** shows mild basal lung infiltrates that progress significantly on December 4th **b** to extensive bilateral consolidations

At admission, the patient was afebrile, had a blood pressure of 152/87, a pulse of 73 beats per minute, a respiratory rate of 16 breaths per minute, and blood oximetry showed a saturation of 96% while breathing room air. The abdominal exam was relatively unremarkable with a negative Murphy’s sign and absence of defense or rebound tenderness. An abdominal CT was ordered and showed fat infiltration around a distended gallbladder. The latter also had wall thickening and stones. These findings were compatible with acute cholecystitis (Fig. 1). However, at the right lung base, subsegmental ground glass opacity was also identified. SARS-CoV-2 could not be detected by quantitative reverse transcription polymerase chain reaction (RT-qPCR) from an oro-nasopharyngeal swab done at admission. The patient underwent diagnostic bronchoscopy the next day and the bronchoalveolar lavage (BAL) was positive for SARS-CoV-2 with cycle threshold (Ct) values of 20.64, 24.01 and 22.00 for the E, RdRp and N genes respectively by the Seegene Allplex 2019-nCoV assay (Seoul, South Korea) (Fig. 2). The following day, an oro-nasopharyngeal swab was once again negative (Fig. 2). The patient had a fever of 38.9 °C on December 2nd and Piperacillin-Tazobactam was initiated. His C-reactive protein levels were at 185 mg/L. He also experienced transient auricular fibrillation, hypotension and his oxygen saturation levels dropped to 92%. He was treated with 150 mg of intravenous Amiodarone. An urgent cholecystectomy drain was placed and the patient was transferred to the ICU requiring 6  L/min of O2 to maintain a saturation of 94%. Chest X-ray on December 4th showed a rapid increase in alveolar lung infiltrates bilaterally when compared to an X-ray 2 days prior (Fig. 3). Decadron was administered for covid as well as Lasix for possible lung edema. A salivary sample was sent for testing and came back positive with a Ct of 36.45 for the N gene exclusively. The following day, another salivary test was performed and the viral load had significantly increased to a Ct value of 24.49 for the N gene (Fig. 2). Successive nasopharyngeal and salivary tests were performed. The values are presented in Fig. 2. On the 4th of December, the patient’s oxygen requirements reached 10  L/min and then slowly improved from there. Piperacillin-tazobactam was stopped on December 9th. The details of the patient’s testing and blood work results, as well as the drugs he received, can be found in the Additional file 1: Tables S1–S3.

## Discussion and conclusions

This patient was admitted with cholecystitis and was incidentally found to have a right lower lobe localized focus of COVID-19 pneumonia in the absence of respiratory symptoms (Fig. 1). The patient was tested for the presence of SARS-CoV-2 in his nose and oropharynx (swab), saliva (spit) and/or right lung (bronchoalveolar lavage) throughout his hospitalization and as symptoms appeared (Fig. 2).

Our results suggest that the primary site of infection was the LRT. In fact, a high viral load (Ct < 24) was detected by RT-qPCR in the BAL on the December 1, while oro-nasopharyngeal swabs on November 30 and December 2 were both negative (Fig. 2). Moreover, the extremely low viral loads detected in the saliva on the December 3rd (mostly below the detection limit), followed by a sharp increase on December 4th and stability throughout the rest of his stay, indicates very early signs of infection of the URT on day four of hospitalization. Compared to the burgeoning infection in the right lung at that time, it seems very unlikely that viruses could have actively replicated in the URT before contaminating the right lung and still be undetectable. Therefore, we reject the possibility that the virus could be found in the patient’s URT on his first days at the hospital. The fact that the patient’s saliva and oro-nasopharyngeal swabs became positive to SARS-CoV-2 during his stay also refutes the possibility of prior replication in the URT, followed by complete clearing.

The sharp increase in saliva titers is likely explained by the development of a secondary replication site in the URT as the illness progressed. We cannot rule out that the virus may have only been deposited from the LRT by exhalation and migration of pulmonary secretions. However, considering the presence of the receptor for SARS-CoV-2 (ACE2) all throughout the respiratory tract [17–19], it seems very unlikely that no virus would infect the URT if deposited on the mucosa. The infection may have progressed to the nasopharynx during days 4 to 8 of hospitalization, since oro-nasopharyngeal swabs were positive on December 8, but still at a lower viral load than saliva samples (Fig. 2). Viruses may have been mostly replicated in the oropharynx and found in the saliva at that time or this difference of signal intensity between the swabs and the saliva samples may be explained by the nature of the sampling procedures and their respective recuperation rates.

We conclude that the infection migrated from the LRT to the URT as the illness progressed and the patient started to develop symptoms. This was also accompanied by the diffusion of the infection in the right and left lung, as shown in Fig. 3. The possibility that the bronchoscopy played a role in transporting the virus to the URT while the apparatus was removed cannot be excluded. It could also have induced the dissemination to the left lung, even if the bronchoscope did not enter it. However, since many patients develop multifocal pneumonia without ever getting a bronchoscopy [20], we believe this to be less likely. We hypothesize that aerosols produced in the primary replication site became trapped in the bronchial and tracheal dead spaces upon expiration and then disseminated to other regions of the lung during subsequent inhalation, explaining the rapid progression from a single focus of infection to bilateral infiltrates.

In order for this patient to have a single pulmonary focus of infection without initial upper respiratory virus replication, he must have inhaled virus-containing respirable aerosol particles in his community. Small respirable particles are far more likely to deposit so deep into the respiratory tract, although all particle size may reach the lung regions with variable probabilities, according to models [21]. However, when inhalable aerosols are generated by coughing, primary LRT infections seem statistically less prone to happen than in the URT [16].

This case gives indirect but relatively compelling evidence that respirable aerosols may spread the virus and cause pneumonia by direct impaction in the lungs rather than the usual seeding from upper respiratory tract replication. The proofs presented in this case are not sufficient by themselves to claim without a doubt the capability of the virus to transmit by aerosols. On the other hand, when combined with reports of SARS-CoV-2 dissemination in the environment through air and epidemiological studies suspecting transmission by air [22–25], the possible role of respirable aerosols in the transmission of COVID-19 seems appreciable.

We acknowledge that the patient was first seeking treatment for cholecystitis, which included multiple drugs (Additional file 1: Tables S3), and that his condition and treatment could have influenced the way the SARS-CoV-2 infection progressed. Yet, it is very improbable that a primary case of COVID-19 pneumonia could have been studied as early as in this report without the presence of another thoracic or abdominal condition, since patients that are not suspected of having COVID-19, who do not show respiratory symptoms early on, and that test negative from URT sampling, would probably never undergo chest X-ray and bronchoscopy.

The fact that some patients test positive for nasopharyngeal swabs and not for saliva, or vice versa*,* may be caused by the localization of the initial impaction and the primary viral replication site along the respiratory tract. The way a patient first comes in contact with the virus might explain his or her own viral replication dynamics and migrations. Since it has been acknowledged that the virus can also transmit by larger particles (droplets) that [26], if inhaled, would most likely impact in the URT [16], multiple scenarios could lead to infection.

It remains unclear if the localization of the first replication site could have an influence on the clinical outcomes. However, since critical cases of COVID-19 imply acute respiratory syndromes, a lung condition, the timing in which the virus reaches the lung may be of importance and requires investigation.

The article presents a case of primary COVID-19 pneumonia. It highlights the possibility of aerosol transmission and the limitation of upper respiratory tract-based detection.

## Supplementary Information

**Additional file 1**:** Table S1.** Ct values of the SARS-CoV-2 tests throughout the patient’s hospitalization.** Table S2.** Blood work results throughout the patient’s hospitalization**. Table S3.** Administered drugs throughout the patient’s hospitalization.

## Data Availability

All data are available in the additional materials.
