# Supplementary material for: A case of primary COVID-19 pneumonia: plausible airborne transmission of SARS-CoV-2
Source: Eur J Med Res. 2022 Apr 4;27:50. doi: 10.1186/s40001-022-00668-1 (PMC8977185; doi:10.1186/s40001-022-00668-1)
Supplement: Supplementary file 1 — Additional file 1: Table S1. Ct values of the SARS-CoV-2 tests throughout the patient’s hospitalization. Table S2. Blood work results throughout the patient’s hospitalization. Table S3. Administered drugs throughout the patient’s hospitalization. [file 40001_2022_668_MOESM1_ESM.docx]

Additional materials for the case report:

**A case of primary COVID-19 pneumonia: plausible** **airborne transmission of SARS-CoV-2**

**AUTHORS**

Nathan Dumont-Leblond^1^, Caroline Duchaine^1, 2, 3^, Marc Veillette^1^, Visal Pen^4^ and Marco Bergevin^5*^

**AUTHORS’ AFFILIATION**

1. Centre de recherche de l’Institut universitaire de cardiologie et de pneumologie de Québec, Quebec City, QC, Canada
2. Département de biochimie, de microbiologie et de bio-informatique, Faculté des sciences et de génie, Université Laval, Quebec City, QC, Canada
3. Canada Research Chair on Bioaerosols, Quebec City, QC, Canada
4. Cité-de-la-Santé Hospital, Department of Medical Imaging, Laval, QC, Canada
5. Cité-de-la-Santé Hospital, Department of Microbiology, Laval, QC, Canada

*Corresponding author E-mail: marco-andres.bergevin.med@ssss.gouv.qc.ca

This document includes:

**Additional Tables S1-S3**

| Day | Ct values | | | | | | | | |
| --- | --- | --- | --- | --- | --- | --- | --- | --- | --- |
|  | BAL  E gene | BAL  RdRp  gene | BAL  N gene | Nasopharyngeal swab  E gene | Nasopharyngeal swab  RdRp gene | Nasopharyngeal swab  N Gene | Saliva  E gene | Saliva  RdRp gene | Saliva  N Gene |
| 30-Nov |  |  |  | Not detected | Not detected | Not detected |  |  |  |
| 01-Dec | 20.64 | 24.01 | 22.00 |  |  |  |  |  |  |
| 02-Dec |  |  |  | Not detected | Not detected | Not detected |  |  |  |
| 03-Dec |  |  |  |  |  |  | Not detected | Not detected | 36.45 |
| 04-Dec |  |  |  |  |  |  | 21.63 | 26.18 | 22.45 |
| 05-Dec |  |  |  |  |  |  |  |  |  |
| 06-Dec |  |  |  |  |  |  |  |  |  |
| 07-Dec |  |  |  |  |  |  | 30.57 | 34.21 | 24.49 |
| 08-Dec |  |  |  | Not detected | Not detected | 32.12 |  |  |  |
| 09-Dec |  |  |  |  |  |  | 23.74 | 27.05 | 26.06 |
| 10-Dec |  |  |  |  |  |  |  |  |  |
| 11-Dec |  |  |  | 34.46 | 37.88 | 36.86 | 27.43 | 31.62 | 29.61 |
| 12-Dec |  |  |  |  |  |  |  |  |  |

**Additional Table S1: Ct values of the SARS-CoV-2 tests throughout the patient’s hospitalization**

**Additional Table S2: Blood work results throughout the patient’s hospitalization^*^**

*Values in red are considered abnormal

| Day | WBC 10e^9^/L  [4.2-11.0] | Lymphocytes 10e^9^/L  [1.0-4.0] | Neutrophils 10e^9^/L  [1.8-7.0] | Platelets 10e^9^/L  [140-440] | Hb g/L  [134-175] | CRP mg/L  [0-8] | Creatinine μmol/L  [59-104] | ALT U/L  [13-50] | Hydroxy D  nmol/L  [75-125] | Ferritin  μg/L  [26.0-388.0] | HbA1G  %  [4.0-6.0] | Blood culture |
| --- | --- | --- | --- | --- | --- | --- | --- | --- | --- | --- | --- | --- |
| 30-Nov | 8.8 | 1.04 | 6.77 | 194 | 159 |  | 47 | 41 |  |  | 8.4 |  |
| 01-Dec | 16.4 |  |  | 170 | 159 |  |  |  |  |  |  |  |
| 02-Dec | 12.4 | 0.74 | 11.01 | 125 | 144 | 185 | 51 | 31 |  |  |  | Negative |
| 03-Dec | 9.2 |  |  | 123 | 138 |  | 47 |  |  |  |  |  |
| 04-Dec | 9.2 | 0.3 | 10.42 | 110 | 138 | 157 | 40 |  |  |  |  |  |
| 05-Dec | 12.4 |  |  | 186 | 142 |  | 47 |  |  |  |  |  |
| 06-Dec | 9 |  |  | 265 | 155 | 128 | 42 |  |  |  |  |  |
| 07-Dec | 8.8 | 1.28 | 6.53 | 275 | 149 |  | 47 |  |  |  |  |  |
| 08-Dec | 8.8 | 1.87 | 5.91 | 359 | 155 |  | 45 | 76 |  |  |  |  |
| 09-Dec |  |  |  |  |  |  | 43 |  |  |  |  |  |
| 10-Dec | 15.1 | 2.07 | 11.94 | 529 | 159 |  | 41 | 87 |  |  |  | 1/4  *S. hominis* |
| 11-Dec | 15.6 | 2.82 | 11.5 | 602 | 149 |  | 48 | 82 | 35 | 1489.2 |  |  |
| 12-Dec | 16.9 | 3.6 | 11.98 | 747 | 146 | 6 | 50 | 72 |  |  |  |  |

**Additional Table S3 : Administered drugs throughout the patient’s hospitalization**

| Day | Piperacillin-Tazobactam  (Tazocin) | Dexamethasone  (Decadron) | Amiodarone | Lovenox | Furosemide (Lasix) |
| --- | --- | --- | --- | --- | --- |
| 30-Nov |  |  |  |  |  |
| 01-Dec |  |  | 150mg IV |  |  |
| 02-Dec | 3.375 g IV q 6h |  |  |  |  |
| 03-Dec | 3.375 g IV q 6h | 6 mg IV qd |  | 40/mg subcutaneous |  |
| 04-Dec | 3.375 g IV q 6h | 6 mg po qd |  | 40/mg subcutaneous | 60mg IV |
| 05-Dec | 3.375 g IV q 6h | 6 mg po qd |  | 40/mg subcutaneous |  |
| 06-Dec | 3.375 g IV q 6h | 6 mg po qd |  | 40/mg subcutaneous |  |
| 07-Dec | 3.375 g IV q 6h | 6 mg po qd |  | 40/mg s subcutaneous |  |
| 08-Dec |  | 6 mg po qd |  | 40/mg subcutaneous |  |
| 09-Dec |  | 6 mg po qd |  | 40/mg subcutaneous |  |
| 10-Dec |  | 6 mg po qd |  | 40/mg subcutaneous |  |
| 11-Dec |  | 6 mg po qd |  | 40/mg subcutaneous |  |
| 12-Dec |  | 6 mg po qd |  | 40/mg s subcutaneous |  |
